# Dipole-Driven Charge Trapping in Monolayer Janus MoSSe for Ultrathin Nonvolatile Memory Devices

**DOI:** 10.1007/s40820-026-02078-y

**Published:** 2026-01-26

**Authors:** Eun Bee Ko, Junho Sung, Seon Yeon Choi, Yasir Hassan, Jeong-Ju Bae, Jongseok Kim, Hyun You Kim, Eunho Lee, Min Sup Choi, Hyun Ho Kim

**Affiliations:** 1 School of Materials Science and Engineering, Kumoh National Institute of Technology, Gumi, 39177 Republic of Korea; 2 Department of Chemical and Biomolecular Engineering, Seoul National University of Science and Technology, Seoul, 01811 Republic of Korea; 3 Department of Materials Science and Engineering, Chungnam National University, Daejeon, 34134 Republic of Korea; 4 School of Materials Science and Engineering, Gwangju Institute of Science and Technology, Gwangju, 61005 Republic of Korea

**Keywords:** Janus TMDs, Nonvolatile memory, Floating-gate, 2D materials, Synaptic device

## Abstract

The continued scaling of flash memory technologies faces challenges such as limited operation speed, poor data retention, and interface defects inherent to conventional three-dimensional architectures. Two-dimensional (2D) materials, with van der Waals interfaces and atomic-scale thickness, offer a promising pathway to overcome these limitations by enabling efficient charge modulation while minimizing surface defects. In this work, a nonvolatile 2D flash memory device is developed employing monolayer Janus MoSSe as the charge-trapping layer and hexagonal boron nitride (h-BN) as an ultrathin tunneling barrier. The intrinsic structural asymmetry of Janus MoSSe induces a strong vertical dipole moment, resulting in enhanced charge trapping, deeper energy barriers, and directional polarization compared with symmetric 2D materials. Consequently, the devices exhibit outstanding retention times exceeding 10^4^ s, endurance beyond 10^4^ program/erase cycles, and large memory window ratios (Δ*V*/*V*_G,max_ of 50%–70% for 10 and 6 nm h-BN, respectively), with charge-trapping rates up to 8.96 × 10^14^ cm^−2^ s^−1^. In addition, Janus MoSSe-based devices show synaptic characteristics under electrical pulses and perform recognition simulations in artificial neural networks. These findings establish a design paradigm for 2D memory devices, enabling ultrathin, flexible, and energy-efficient nonvolatile memories.

**Supplementary Information:**

The online version contains supplementary material available at 10.1007/s40820-026-02078-y.

## Highlights

- Janus MoSSe-based floating-gate memory exhibits ultrafast charge-trapping dynamics and stable charge retention exceeding 10^8^ s under low-voltage operation.
- The intrinsic out-of-plane dipole moment in Janus MoSSe effectively suppresses leakage current and enlarges the memory window, even with ultrathin h-BN tunneling layers.
- The proposed all-van der Waals heterostructure provides a scalable platform for high-speed, energy-efficient, and reliable nonvolatile memory applications.

**Supplementary Information:**

The online version contains supplementary material available at 10.1007/s40820-026-02078-y.

## Graphical abstract

**Figure:**
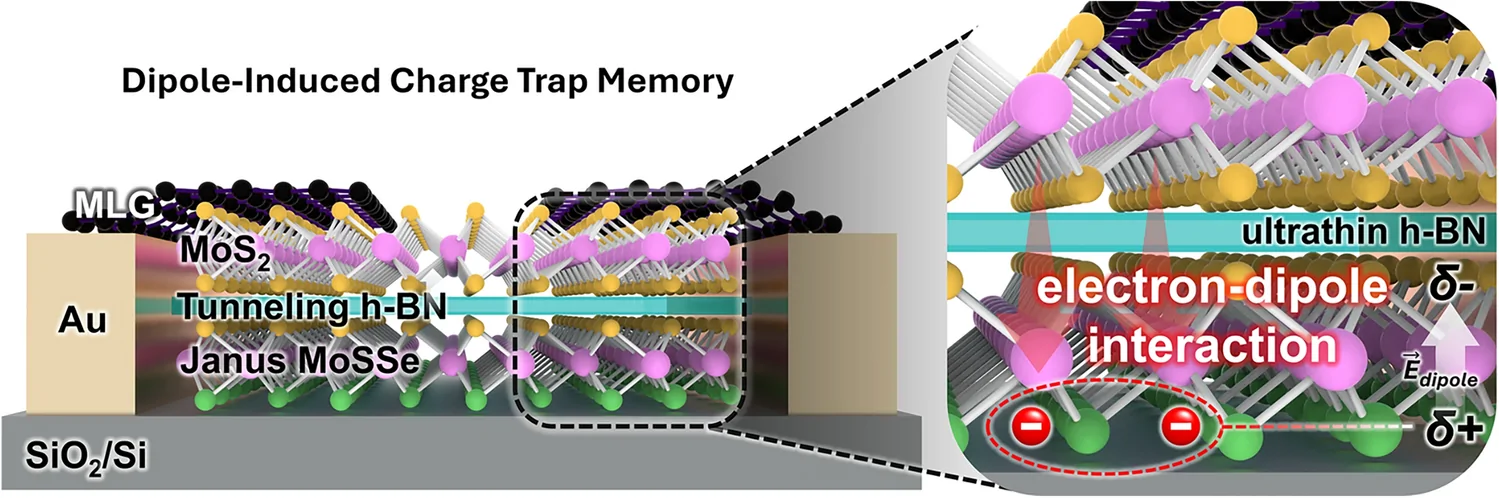

## Introduction

Nonvolatile flash memory devices are key components in the advancement of next-generation electronics, particularly for ultrafast, highly stable, and high-density data storage in the modern semiconductor industry [1]. As digital infrastructure increasingly demands energy-efficient and scalable memory technologies for applications such as flexible electronics [2], wearables, neuromorphic computing [3], and the Internet of Things, the development of high-performance memory devices becomes even more critical [4, 5]. However, conventional 3D flash memory architectures are facing scaling bottlenecks, including low operation speeds, short data retention, limited endurance, and poor interface quality, which stem from issues such as surface dangling bonds, interface roughness, and inefficient charge tunneling [6, 7]. To overcome these limitations, two-dimensional (2D) materials have emerged as highly promising candidates. Their atomic thinness, the absence of surface dangling bonds, and clean van der Waals (vdW) interfaces enable precise electrostatic control, efficient charge modulation, and defect-minimized device behavior [8–11]. These advantages have driven significant research into 2D-material-based memory, logic, and optoelectronic devices [12].

Among the critical design parameters for 2D flash memory devices, the tunneling barrier and the charge trapping layer are decisive factors that govern memory window, retention time, and endurance. Dielectric materials such as hexagonal boron nitride (h-BN) [13], aluminum oxide (Al_2_O_3_) [14], and hafnium oxide (HfO_2_) [15] have been widely employed as tunneling barriers owing to their large bandgap and chemical stability. In parallel, 2D materials including MoS_2_ [13], graphene [16], and WSe_2_ [9] have been explored as charge trapping layers because of their atomic thinness and compatibility with vdW heterostructures. Despite these advances, their performance is often limited by symmetric charge distribution and shallow trapping centers, which result in reduced charge retention, accelerated leakage, and relatively narrow memory windows [17]. These intrinsic drawbacks underscore the urgent need for alternative charge-trapping materials that can provide deeper and more stable trap states, enhanced polarity, and improved electrostatic control.

In this work, we introduce the use of monolayer Janus MoSSe as a charge trapping material in a 2D flash memory device. Janus MoSSe, a unique transition metal dichalcogenide (TMD), possesses a built-in vertical dipole due to its structural asymmetry, with sulfur (S) atoms on one side and selenium (Se) on the other side [18, 19]. This internal electric field not only enhances charge trapping and retention but also introduces directional charge polarization, which is unavailable in conventional symmetric 2D TMDs. The fabricated 2D memory device with Janus MoSSe charge trapping layer exhibits exceptional performance, long-term stability of 10^4^ s, endurance up to 10^4^ program/erase cycles, high memory window, Δ*V*/*V*_G,max_ ratio of 50% (10 nm h*-*BN) and 70% (6 nm h-BN) at a charge trapping rate of 2.97 × 10^14^ cm^−2^ s^−1^ for the 10-nm-thick h-BN device and 8.96 × 10^14^ cm^−2^ s^−1^ for the 6-nm-thick h-BN device, compared to previously reported 2D-material-based flash memories. Janus MoSSe with intrinsic dipole enables deeper and stable trapping states and higher energy barriers, resulting in superior charge retention and reduced charge leakage. Asymmetric charge transport arising from the alignment between the intrinsic dipole and the external gate electric field leads to a preferential, one-sided expansion of the memory window. The combination of h-BN and Janus MoSSe ensures both efficient tunneling and robust charge confinement, a balance rarely achieved in prior systems. Finally, the fabricated device successfully achieved synaptic weight updates such as paired-pulse facilitation (PPF), long-term plasticity (LTP), and long-term potentiation/depression (LTP/D). We also achieved high accuracy in artificial neural network (ANN)-based simulations reflecting the obtained device characteristics. This study establishes a new design paradigm for 2D flash memory devices by leveraging the asymmetric dipolar nature of Janus MoSSe, achieving unprecedented memory performance metrics in a simple and scalable architecture. These findings pave the way for next-generation, ultrathin, high-performance nonvolatile memories with applications in AI hardware, flexible electronics, and low-power logic circuits.

## Experimental Section

### Device Fabrication and Characterization

#### Stamp Preparation

The poly(bisphenol A carbonate) (PC) film was prepared by dissolving it in chloroform at a concentration of 0.15 g mL^−1^. The solution was drop-cast onto a glass slide to form a film, which was subsequently dried. The dried PC film was then attached to a polydimethylsiloxane (PDMS, PF-40/17-X4, Gel-Pak) block, and the PDMS block was fixed onto a glass slide to serve as a stamp.

#### Device Fabrication

The heterostructured memory devices were fabricated inside a nitrogen-atmosphere glovebox (${P}_{{O}_{2}}$, ${P}_{{H}_{2}O}$ < 0.1 ppm) equipped with a custom-built microscope/transfer system. The process began by picking up graphene, which served as the contact electrode. Subsequently, the channel layer (MoS_2_) was aligned and picked up, followed by sequential pickup of the tunneling layer (h-BN) and the floating-gate layer (Janus MoSSe). Detailed procedures for the pickup process have been described previously [20]. The assembled stack was then transferred onto a pre-patterned Ti/Au (3/30 nm) electrode deposited on a p-type Si substrate with a 285-nm SiO_2_ layer (Silicon Technology Co.). After transfer, the supporting PC film was removed using chloroform.

#### Characterization

Micro-Raman spectroscopy (UniRAM, UniNanoTech) with a 532-nm excitation laser was employed to characterize MoSe_2_ and Janus MoSSe grown on SiO_2_/Si substrates. Electrical measurements were performed at room temperature in ambient conditions using two DC source meters (Keithley 2450) with needle-type probes. The thickness of 2D materials, including Janus MoSSe, was measured in tapping mode using atomic force microscopy (FX40, Park Systems).

### Simulation and Computational Methods

#### Simulation Parameter

The dynamic range was obtained from the on/off ratio of the LTP/D curves, while *NS*_eff_ was defined as the number of valid conductance states exceeding a noise level, set at 0.5% of *G*_max_—*G*_min_. *NL* values were calculated using the following equations [21, 22]:

1 $${\mathrm{G}}_{\mathrm{LTP}}\text{ = B}\left(\text{1 }- \, {\text{e }}^{\left(-\frac{\mathrm{P}}{{\mathrm{A}}_{\mathrm{P}}}\right)}\right)\text{ + }{\mathrm{G}}_{\mathrm{min}}$$

2 $${\mathrm{G}}_{\mathrm{LTD}}\text{ = }-{\mathrm{B}}\left(\text{1 }- {\mathrm{e}}^{\left(\frac{\text{P }-{\text{ P}}_{\mathrm{max}}}{{\mathrm{A}}_{\mathrm{D}}}\right)}\right)\text{ + }{\mathrm{G}}_{\mathrm{max}}$$

3 $$\text{B = }\left({\mathrm{G}}_{\mathrm{max}}-{\mathrm{G}}_{\mathrm{min}}\right) /\text{ (1 }-{\mathrm{e}}^{\frac{-{\mathrm{P}}_{\mathrm{max}}}{{\mathrm{A}}_{\text{P, D}}}}\mathrm{)}$$

where G represents the conductance value in the LTP/D curve, P is the number of applied pulses, and A denotes a fitting parameter governing nonlinearity in EPSC modulation.

#### ANN-based Simulation Details

The ANN simulations were performed on a Linux system utilizing the open-access software MLP + NeuroSimV3.0 based on a multilayer perceptron structure. The ANN was designed with an input layer comprising 784 neurons (corresponding to 28 × 28 pixels), a hidden layer of 100 neurons, and an output layer of 10 neurons representing digits 0–9. The 60,000 images used in training were processed in batches of 8,000 images per epoch with a batch size of 64, and the size of the weights was standardized for maximum = 1 and minimum =  − 1. To consider the characteristics of Janus MoSSe-based devices, we used device characteristic values such as nonlinearity *(NL)* dynamic range, gate/drain voltage, pulse number, and cycle-to-cycle (C2C) and device-to-device (D2D) variation. C2C variation was defined as the percentage term of the conductance range defined as *G*_max_ − *G*_min_. D2D variation was calculated as the standard deviation of *NL* values between different devices, and the larger of the LTP and LTD regions was reflected in the simulation to be conservative.

#### CNN-based Simulation Details

CNN simulations were performed on a Linux system using the open-source program DNN + NeuroSimV2.1 to evaluate the classification accuracy on CIFAR-10 data. For the computation, we adopted a VGG-8 structure consisting of six convolutional/pooling layers and two fully connected layers. During the training and inference process, the images were converted into feature maps (FMs) consisting of the input signals (*V*) and weights (*W*) convolved with the current signal (*I*). The generated FMs were transformed through ReLU activation functions and scaled down and emphasized through max-pooling in the 2nd, 4th, and 6th layers. Learning and inference were then successfully performed through the fully connected layers. To reflect the characteristics of Janus MoSSe-based devices in these computations, device parameters were applied as in the ANN-based simulations.

#### Density Functional Theory Calculations

All spin-polarized DFT calculations were performed using the Vienna ab initio simulation package (VASP) code and the Perdew–Burke–Ernzerhof (PBE) functional [23–25]. The projector augmented wave method describes the interaction between the ionic core and valence electrons [24]. The DFT + U scheme [26], with U_eff_ = 4 eV [27], was applied to the Mo ions to treat the localized Mo-d orbitals appropriately. Valence electron wave functions were expanded on a plane-wave basis up to an energy cutoff of 400 eV. The first Brillouin zone was sampled at the Γ-point for initial geometry optimizations and expanded to a 6 × 6 × 1 grid for further electronic analyses. Convergence criteria for electronic structure and atomic geometries were 10^–4^ eV and 0.05 eV Å^−1^, respectively. A Gaussian smearing function with a finite temperature width of 0.01 eV was employed to enhance the convergence of states near the Fermi level. The initial MoS_2_ motif was adopted from our previous study [28]. To virtually reproduce the overall geometry of the Janus MoSSe floating-gate memory device, we combined two 4 × 4 MoS_2_ layers (top and bottom) and a h-BN interfacial layer. The h-BN-MoS_2_ interlayer spacing was initially set to the literature-reported vdW distance [29] and optimized. The bottom-most S atoms in the bottom MoS_2_ layer were substituted with Se to construct the final Janus MoSSe-h-BN-MoS_2_ model structure (Fig*.* S2). The vacuum layer between the Janus MoSSe-h-BN-MoS_2_ triple layers was set to 10 Å.

## Results and Discussion

### Structural Asymmetry and Charge–Dipole Interaction Mechanism of Janus MoSSe

**Fig. 1:**
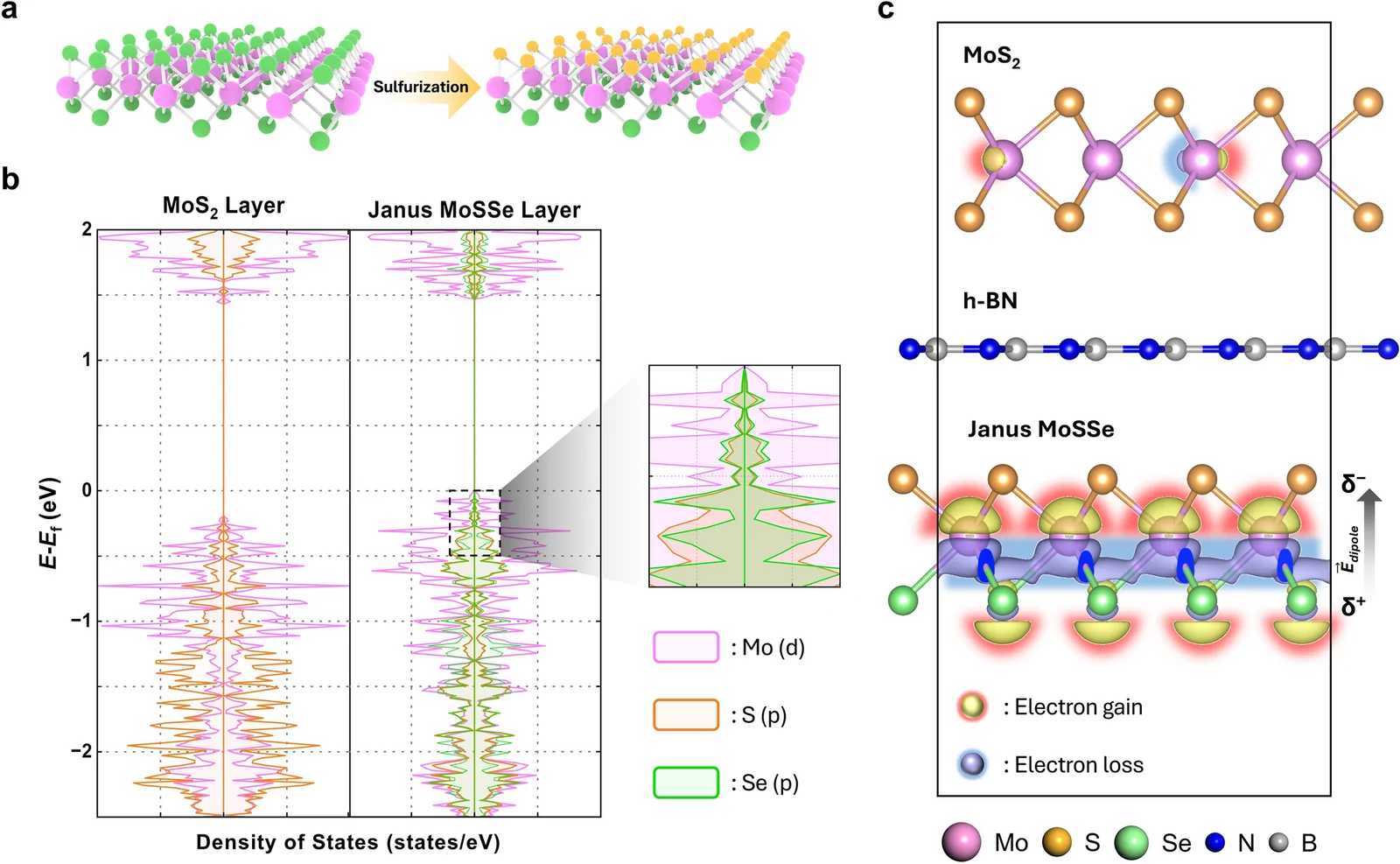
**a** Schematic illustration of the sulfurization process converting MoSe_2_ into Janus MoSSe by substituting the top Se atoms with S atoms. **b** DFT-calculated density of states (DOS) of each MoS_2_ and Janus MoSSe layer, showing a pronounced contribution of Se p-orbitals near the Fermi level in the Janus structure upon electron injection. **c** Charge density difference map of the MoS_2_/h-BN/Janus MoSSe heterostructure, where yellow and blue regions represent the area where electrons are localized or depleted

A novel memory device structure that exploits the built-in dipole moment of Janus TMDs is investigated. Janus TMDs possess an asymmetric atomic configuration, in which the top and bottom chalcogen layers are composed of distinct atomic species. This out-of-plane asymmetry gives rise to a permanent electric dipole, leading to strong charge–dipole interactions that significantly influence the charge trapping behavior in memory devices. The unique atomic configuration and structural characteristics of Janus TMDs are illustrated in Fig. 1a. To realize this structure experimentally, MoSe_2_ was grown using a conventional atmospheric pressure chemical vapor deposition (APCVD) method (Fig. S1a) [30]. Specifically, single-crystalline MoSe_2_ nanosheets were synthesized in an APCVD reactor under an Ar/H_2_ atmosphere, using MoO_3_ and Se powder as precursors. The as-grown MoSe_2_ nanosheets were then converted into Janus MoSSe nanosheets by applying a previously reported plasma-induced sulfurization method (Fig. S1b) [31]. In this process, the top Se atoms were replaced with S under an Ar/H_2_ plasma environment, thereby forming a Janus structure with S on the top layer and Se on the bottom layer. The synthesized Janus MoSSe was examined by optical microscopy (OM) and confirmed to exist as a monolayer by atomic force microscopy (AFM) (Fig. S1c, d). Raman spectroscopy was performed to examine the structural characteristics of the Janus MoSSe. As shown in Fig. S1e, the Raman spectrum of Janus MoSSe exhibits clear peak shifts compared to that of MoSe_2_, indicating successful Janus conversion. Raman mapping analysis further reveals a uniform intensity distribution across the entire Janus MoSSe flake, confirming good spatial uniformity without noticeable degradation (Fig. S1f). In addition, the photoluminescence (PL) spectrum shows a well-defined emission peak, suggesting favorable optical quality of the synthesized Janus MoSSe (Fig. S1g) [30]. Figure S1h further presents the transfer characteristics of a Janus MoSSe transistor, confirming its n-type semiconducting behavior.

Following the verification of the optical characteristics of the synthesized Janus MoSSe, we performed theoretical analysis of the interaction between the built-in dipole moment and electrons in a vdW heterostructure-based floating-gate memory device incorporating Janus TMDs as the floating gate through density functional theory (DFT) calculation. To confirm that the Janus MoSSe captures and localizes excess electrons, we added a total of five electrons (corresponding to 0.5% of total electrons) to the neutral MoS_2_/h-BN/Janus MoSSe heterostructure. We separately present the density of states of each MoS_2_ and Janus MoSSe layer (Fig. 1b) and the corresponding charge density difference map (Fig. 1c), illustrating the orbitals at which the injected electrons are localized. The DOS confirms that the Se *p*-orbitals contribute to the occupied electron states near the Fermi level (Fig. 1b), indicating that Se atoms strongly attract electrons. The charge density difference map of MoS_2_/h-BN/Janus MoSSe heterostructure confirms that injected electrons are primarily localized at Se atoms (yellow area). Furthermore, electron gain (yellow area) and loss (blue area) pairs are accordingly formed around Mo atoms upon electron localization at the Se atoms. The electronic behavior of the MoS_2_/h-BN/Janus MoSSe heterostructure demonstrates that the intrinsic polarity of the Janus structure drives electron localization at Se sites, accompanied by a rearrangement of Mo states. Moreover, the comparison in Fig. S2 between MoS_2_/h-BN/MoS_2_ and MoS_2_/h-BN/Janus MoSSe heterostructures reveals distinct variations in interface distance, bond length, and bond angle, confirming that the structural asymmetry introduced by the Janus layer directly affects both the electronic distribution and the structural characteristics of the heterostructure.

To further elucidate the role of dipole orientation, we additionally performed theoretical calculations on an inverted Janus MoSSe structure, in which the Se-terminated side faces upward (Fig. S3). For the original Janus MoSSe configuration, the injected electrons are primarily stabilized within the Janus MoSSe layer, consistent with effective the charge confinement by the built-in dipole field. In contrast, for the inverted Janus MoSSe structure, the calculated charge density difference map reveals noticeable electron accumulation in the MoS_2_ channel region as well. This redistribution of injected charges toward the channel can be interpreted as a leakage-like behavior, which is expected to degrade charge-trapping stability. These theoretical results indicate that the orientation of the intrinsic dipole plays a critical role in determining charge redistribution and confinement. When combined with the experimentally observed polarity-dependent electrical characteristics, the enhanced Δ*V*_th_ and memory window only under the positive sweep (electron injection) for Janus MoSSe the results consistently support the interpretation that the built-in dipole in Janus MoSSe actively contributes to the charge-trapping behavior. Although a direct experimental visualization of the dipole field is beyond the scope of this work, the qualitative agreement between experiment and theory provides a coherent physical picture for the dipole-assisted charge-trapping mechanism in Janus MoSSe.

### Device Architecture and Memory Characteristics of Janus MoSSe-Based Floating-Gate Memories

**Fig. 2:**
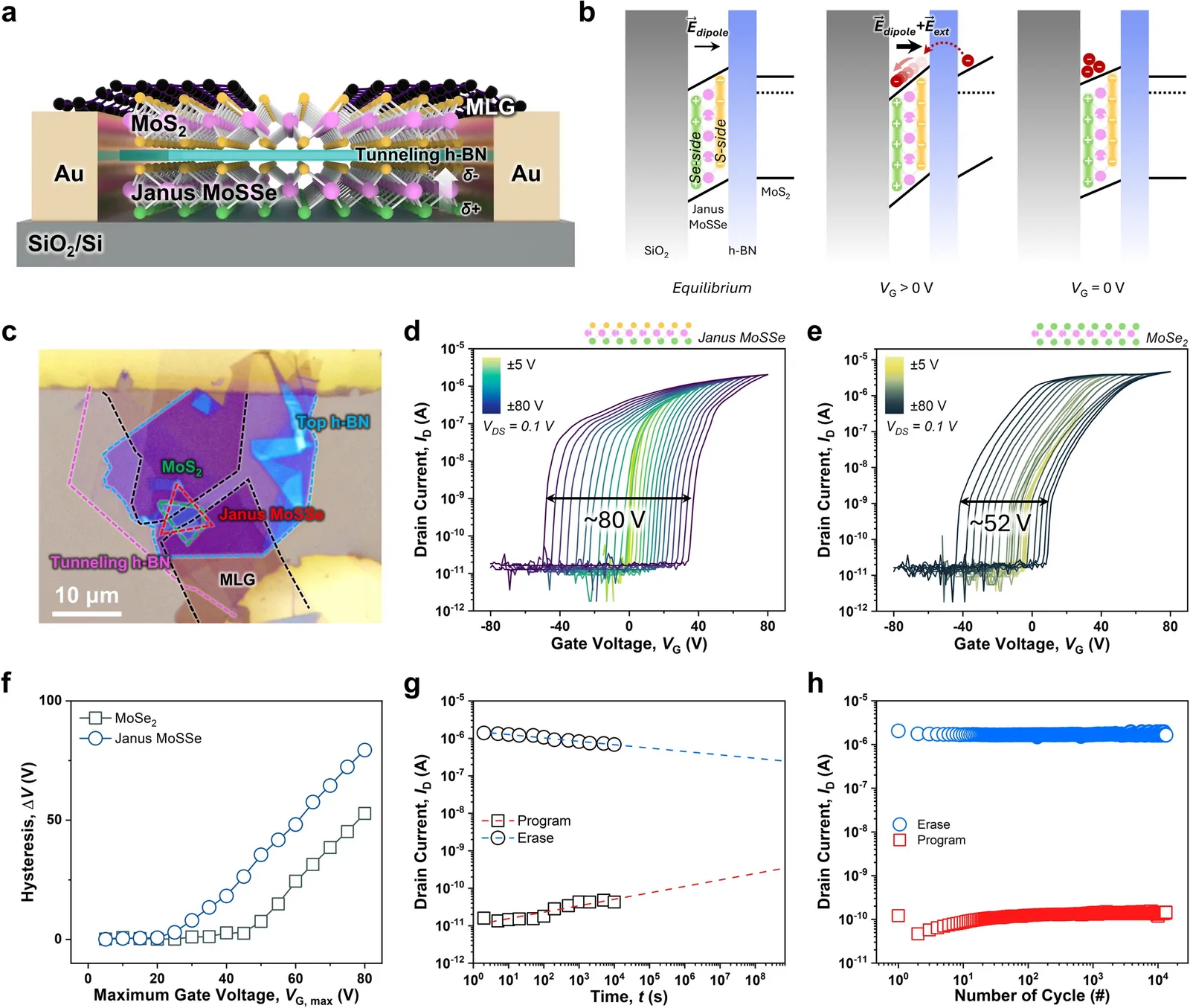
Janus MoSSe floating-gate memory device with 10 nm h-BN as the tunneling layer. **a** Schematic illustration of the vdW heterostructure device. **b** Schematic energy band diagram. **c** OM image of the fabricated device. Transfer curves of **d** Janus MoSSe and **e** MoSe_2_ devices measured under gate sweeps (± 5 ~ 80 V) at *V*_DS_ = 0.1 V. **f** Δ*V* of Janus MoSSe and MoSe_2_ devices as a function of maximum gate voltage. **g** Retention performance measured at *V*_G_ = 0 V with a pulse of ± 80 V, 1 s gate pulses and *V*_DS_ = 0.1 V. **h** Endurance characteristics under repeated program/erase cycling up to 10^4^ cycles

Figure 2a presents a schematic of the 2D flash memory device, in which multilayer graphene (MLG)/MoS_2_/h-BN/Janus MoSSe are vertically stacked on a p-Si/SiO_2_ substrate. Here, p-Si and SiO_2_ function as the control gate and blocking layer, respectively, while Janus MoSSe serves as the floating gate, h-BN as the tunneling layer, MoS_2_ as the channel, and MLG as the source/drain electrodes. Graphene–TMD heterostructures have been utilized for high-performance electronic devices with atomically clean interfaces [32]. As shown in the band diagram of Fig. 2b, the built-in out-of-plane dipole in Janus MoSSe generates an internal electric field (*E*_dipole_). Consequently, when electrons are trapped under an applied gate voltage, they are expected to localize near the Se atoms, as evidenced by prior DFT calculation. This localization of electrons, governed not only by the electric field (*E*_ext_) from the gate voltage but also by the inherent dipole field, enables more effective tunneling and charge storage, thereby enlarging the memory window (Δ*V*) and prolonging charge retention. In contrast, the Janus MoSSe-based structure proposed in this study is fundamentally distinct from previously reported 2D NVMs in that it does not rely on defect-mediated mechanisms [33] or structural phase transitions [34], but instead directly exploits the intrinsic out-of-plane built-in dipole of Janus MoSSe in the charge storage and injection processes. This inherent dipole asymmetrically modulates the tunneling barrier depending on its alignment with the external gate field, thereby facilitating electron injection while suppressing charge release. As a result, enhanced charge-trapping characteristics can be achieved without artificial trap engineering. Such dipole-engineered operation represents a previously unreported mechanism in 2D material-based memory devices and demonstrates the feasibility of a flash memory architecture that directly employs Janus MoSSe as a floating gate.

Figure 2c shows the OM image of an actual fabricated device. Other 2D materials employed in this study, excluding Janus MoSSe, were mechanically exfoliated and subsequently stacked to form vdW heterostructures (see Methods and Fig. S4 for detailed fabrication steps). Figure 2d shows the transfer characteristics measured under different gate-voltage sweep conditions. The drain voltage (*V*_DS_) was fixed at 0.1 V, while the control gate voltage (*V*_G_) was swept between ± 80 V. A pronounced hysteresis window was observed, confirming the nonvolatile memory operation of Janus MoSSe floating-gate devices. For comparison, a control device employing MoSe_2_ as the floating gate, with an otherwise identical structure, exhibited a relatively small hysteresis window under the same measurement conditions (Fig. 2e). As shown in Fig. 2f, the memory window of both devices increased proportionally with the maximum swept gate voltage (*V*_G,max_). At *V*_G,max_ =  ± 80 V, Janus MoSSe-based device exhibited a memory window of Δ*V* = 79.4 V, approximately 1.5 times larger than that of the MoSe_2_-based device (Δ*V* = 52.8 V). Compared with the MoSe_2_-based device, the Janus MoSSe-based device exhibits a more pronounced increase in the memory window in the positive gate-voltage direction. This behavior can be attributed to the intrinsic structural asymmetry of Janus MoSSe and its associated dipole moment, which enhances charge trapping when aligned with the applied positive gate electric field. This enhancement arises from the inherent structural asymmetry and built-in dipole moment of Janus MoSSe, which promote more efficient charge trapping compared to symmetric TMDs.

To evaluate memory reliability, endurance and retention characteristics of Janus MoSSe-based devices were investigated. As shown in Fig. 2g, the drain current (*I*_D_) was monitored at *V*_G_ = 0 V over time with program/erase pulses of ± 80 V for 1 s duration. Both program and erase states remained highly stable up to 10^4^ s, and extrapolation predicts data retention exceeding 10 years. Retention measurements at elevated temperatures of 85 and 100 ℃ further confirm the thermal stability of the stored charges, showing negligible variation in both programmed and erased states (Fig. S5a). Furthermore, endurance cycling tests (Fig. 2h), performed using ± 80 V program/erase pulses with a pulse width of 30 ms, demonstrated reliable operation up to ~ 10^4^ program/erase cycles without significant degradation, confirming the robust memory characteristics of Janus MoSSe floating-gate devices. To examine faster operation, the device was also tested using shorter program/erase pulses down to 500 ns, under which clear and stable program and erase states were still observed (Fig. S5b).

The data depicted in Fig. 2 correspond to devices integrating an h-BN tunneling layer with a thickness of approximately 10 nm. Decreasing the h-BN layer thickness enhances electron tunneling probability, thereby facilitating faster program/erase operations and enabling the development of high-speed memory devices. Nonetheless, excessively thinning the h-BN layer results in increased leakage current, which adversely affects retention performance. Conversely, increasing the thickness of h-BN suppresses electron tunneling, leading to slower program/erase responses and a narrower memory window, yet effectively diminishes leakage current, thus promoting improved long-term data retention and device reliability [17, 35]. Owing to these inherent trade-offs, prior studies have established that an h-BN layer thickness of approximately 10 nm achieves an optimal compromise between tunneling efficiency and retention properties [7, 35, 36]. To further validate this proposed optimum and enhance device performance, the thickness of the tunneling layer was systematically varied from 4 to 16 nm to compare their transfer characteristics (Fig. S6). Notably, the Janus MoSSe floating-gate memory exhibited the largest memory window at an h-BN thickness of about 6 nm (Fig. S7a). This shift in the optimal tunneling layer thickness can be attributed to the presence of the intrinsic out-of-plane dipole in Janus MoSSe. Unlike conventional floating-gate systems, the built-in dipole field in Janus MoSSe enhances electron injection while simultaneously suppressing charge leakage, even when the tunneling barrier is relatively thin. As a result, efficient charge trapping can be maintained at a reduced h-BN thickness of ~ 6 nm, leading to a larger memory window compared to thicker tunneling layers. This behavior highlights the distinct role of the Janus dipole in modifying the conventional trade-off between tunneling efficiency and retention performance. In addition, a weak current suppression is observed in the transfer curves of the Janus MoSSe device with a 6-nm h-BN tunneling layer. This behavior is attributed to dipole-induced electrostatic modulation of the channel by trapped charges in the Janus MoSSe floating gate, rather than charge leakage, as the memory window continues to increase beyond this region.

Furthermore, devices incorporating bilayer Janus MoSSe as the floating gate were also fabricated and evaluated. The bilayer device exhibits a slightly increased memory window compared to the monolayer-based device; however, the overall performance characteristics remain similar (Fig. S8).

### Effect of h-BN Tunneling Layer Thickness on Charge-Trapping and Retention Behavior

**Fig. 3:**
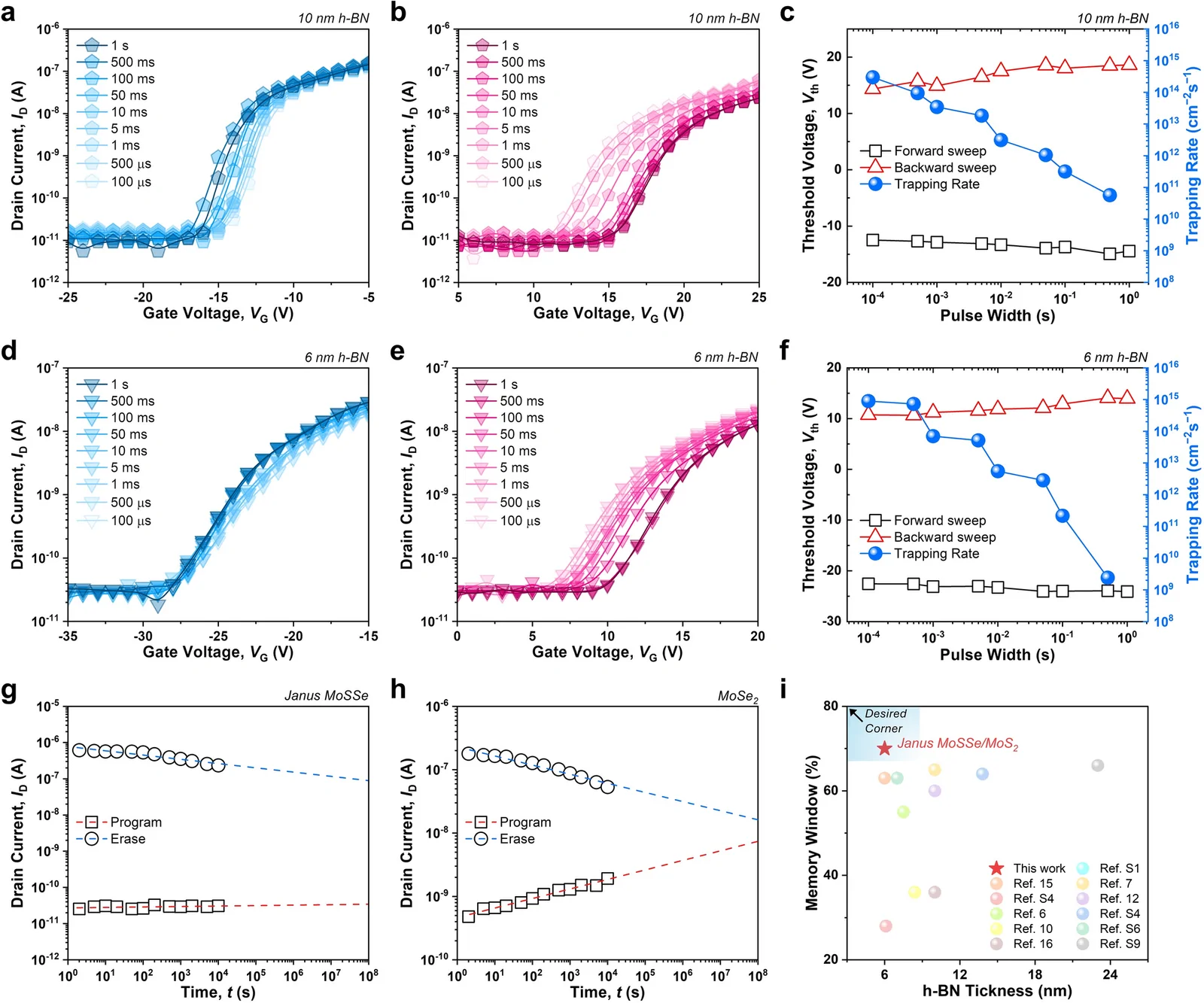
Memory characteristics of Janus MoSSe and MoSe_2_ floating-gate memory devices with different h-BN tunneling layer thicknesses. **a–c** Janus MoSSe memory devices with a 10-nm h-BN tunneling layer, and **d–f** devices with a 6-nm h-BN tunneling layer. **a, d** Transfer characteristics measured after applying − 80 V gate pulses with pulse widths from 1 s to 100 μs, read by a − 40 to 40 V gate sweep; **b, e** Transfer characteristics under + 80 V gate pulses measured under identical read conditions; **c, f** Corresponding threshold voltage shift (Δ*V*_th_) and calculated charge trapping rate. Retention performance of **g** Janus MoSSe and **h** MoSe_2_ memory devices with 6-nm h-BN tunneling layer, measured at *V*_G_ = 0 V with a pulse of ± 80 V (1 s gate pulses) and *V*_DS_ = 0.1 V. **i** Memory window as a function of h-BN thickness for this work and previously reported 2D floating-gate memory devices

In light of these results, Fig. 3 presents a comparative analysis of memory performance between a 6-nm- and 10-nm-thick h-BN tunneling layers. For comparison, the thickness of each of the h-BNs used was verified via AFM (Fig. S9). This approach is predicated on the hypothesis that the intrinsic built-in dipole moment of Janus MoSSe may mitigate the retention loss generally associated with thinner tunneling layers. Figure 3a–c and 3d–f show the program/erase characteristics of Janus MoSSe floating-gate memory devices employing 10-nm- and 6-nm-thick h-BN tunneling layers, respectively, under varying pulse widths. Figure 3a, d shows forward transfer curves from − 40 to 40 V after applying an erasing voltage of − 80 V with different widths. In contrast, Fig. 3b, e shows backward curves after applying a programming voltage of + 80 V with different widths. Each transfer curve was obtained after an independently applied program or erase pulse followed by a gate-voltage sweep, ensuring that all data represent independent measurements without cumulative or history effects. Prolonged pulse durations promote threshold voltage (*V*_th_) modulation due to increased charge trapping, resulting in an enlarged memory window. Figure 3c, f further illustrates *V*_th_ shifts and corresponding charge trapping rates as a function of programming pulse width, as described by the following equations:

4 $$\frac{{dN_{trap} }}{dt} = \frac{{C_{total} }}{e} \cdot \frac{{dV_{G} }}{dt}, dV_{G} /dt \approx \frac{{\Delta V_{th} }}{\Delta t}$$

Here, ${C}_{total}$ represents the sum of the capacitances of SiO_2_, h-BN, and Janus MoSSe,* e* is the elementary charge, Δt is the pulse width, and ${\Delta V}_{th}$ denotes the threshold voltage shift induced by programming pulse [13]. Using this approach, the calculated charge trapping rate for the 6-nm-thick h-BN device was 8.96 × 10^14^ cm^−2^ s^−1^ at pulse width of 100 μs, which is approximately three times higher than that of the 10-nm-thick h-BN device (2.97 × 10^14^ cm^−2^ s^−1^). Notably, the Janus MoSSe-based device maintained sufficient threshold voltage shifts and high trapping rates even under short pulse widths, with these characteristics more pronounced at the 6-nm h-BN thickness. This demonstrates that reducing the h-BN thickness significantly increases electron tunneling probability, thereby enhancing program/erase speeds, and also suggests that the built-in dipole structure of Janus MoSSe enables efficient charge trapping and storage even with thin dielectric layers.

In contrast, MoSe_2_-based devices exhibited a reduced memory window as the h-BN thickness decreased (Fig. S7) The charge trapping rate for the 10-nm-thick h-BN device was 2.12 × 10^14^ cm^−2^ s^−1^, which decreased to 3.34 × 10^13^ cm^−2^ s^−1^ at 6 nm, representing an approximately 6.4-fold reduction (Fig. S10). These results suggest that thinner h-BN layers detrimentally affect charge trapping efficiency in MoSe_2_-based devices. At 6-nm h-BN thickness, the charge trapping rate in the MoSe_2_ device was approximately 26.8 times slower than that of the Janus MoSSe device. This degradation is mainly attributed to the absence of a built-in dipole moment in MoSe_2_, which limits its ability to suppress leakage current and stabilize charge storage under ultrathin tunneling layer conditions. This highlights the critical role of the intrinsic built-in dipole moment in Janus TMDs, which significantly enhances charge trapping performance with ultrathin dielectric thicknesses.

Figure 3g, h compares the retention characteristics of Janus MoSSe and MoSe_2_ floating-gate memory devices using a 6-nm h-BN tunneling layer. Janus MoSSe exhibited long-term stability with a gradual decrease in *I*_D_, retaining 76.5% of its initial on/off ratio (from 4.38 to 3.35 decades after 10 years) similar to the behavior observed at 10 nm h-BN. In contrast, MoSe_2_-based devices showed a rapid current decrease, with the on/off ratio decreasing from 2.57 to 0.19 decades, corresponding to only 7.4% retention. This contrast demonstrates that the built-in dipole in the Janus structure provides deeper and more stable charge trapping sites due to localized electrons in Se atoms, thereby enhancing retention performance.

Figure 3i compares the memory window as a function of h-BN thickness between this work and previously reported 2D floating-gate memory devices. We found that our Janus MoSSe floating-gate devices achieved a record memory window at a tunneling barrier thickness of 6 nm. Compared with Janus MoSSe devices, MoSe_2_ floating-gate devices exhibited an overall smaller memory window, with a particularly pronounced decline at the 6 nm thickness (Fig. S7). The extracted memory window values and device characteristics are summarized in Table S1 for quantitative comparison. These results highlight that the structural asymmetry and inherent dipole moment in Janus materials are key factors that overcome the limitations of conventional TMD-based memory devices and enable excellent memory performance even with ultrathin dielectric layers.

Our findings are currently limited to the practical application of large-area Janus MoSSe due to manual device fabrication. Nevertheless, it is expected to be sufficiently scalable through the currently reported techniques and studies. In particular, for Janus MoSSe, large-scale synthesis is expected to be possible through the currently developed wafer-scale single crystal growth method of TMD and room-temperature plasma substitution [37]. Furthermore, the h-BN used as a tunneling layer in this study is sufficiently replaceable with ALD-depositable Al_2_O_3_ and HfO_2_ [38]. In this respect, our Janus MoSSe memory shows inherent scalability potential.

### Synaptic Plasticity and Neuromorphic Computing Performance of Janus MoSSe Memory Devices

**Fig. 4:**
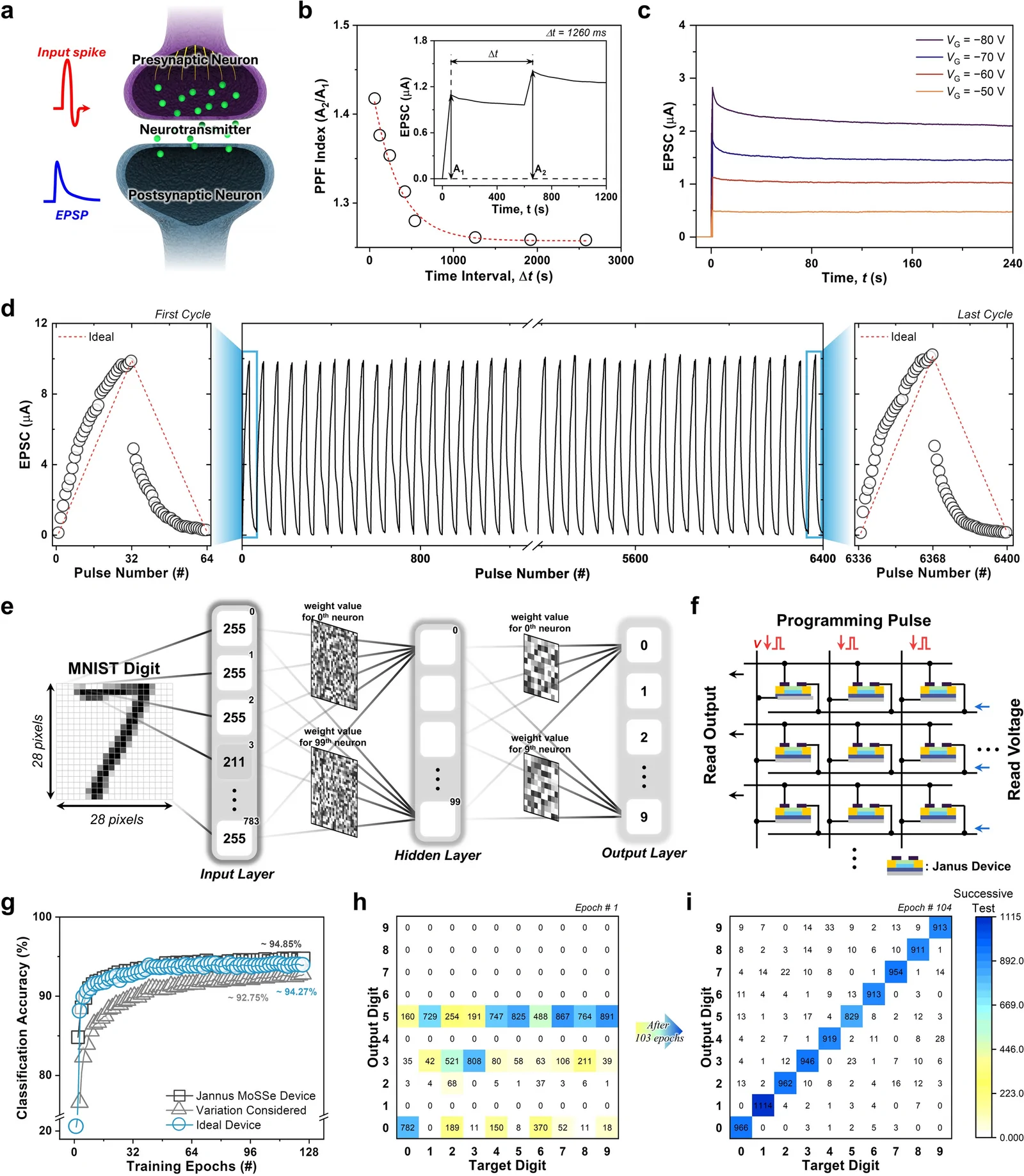
Neuromorphic computing applications of Janus MoSSe-based devices. **a** A unit of signal transmission in the biological nervous system consisting of pre- and postsynaptic neurons and neurotransmitters. **b** The variation over time interval (Δ*t*) of the PPF index (A_2_/A_1_), defined as the ratio of the response to the first pulse (A_1_) to the response to the second pulse (A_1_) for a pair of consecutively applied pulses. Inset: EPSC response when the Δ*t* is 1260 ms. **c** Long-term plasticity characteristics following the application of 10 pulses with different amplitudes (*V*_pulse_ =  − 80, − 70, − 60, and − 50 V). **d** LTP/D curve of a Janus MoSSe device under 64 pulses and LTP/D characteristics change over 100 cycles. **e** Multilayer perceptron consisting of input, hidden, and output layers for ANN operations. **f** Schematic diagram of synaptic array with Janus MoSSe devices and circuits. **g** Recognition accuracy of Ideal device and Janus MoSSe device and D2D and C2C variation considered Janus MoSSe devices over 125 epochs. Confusion matrix for recognition accuracy of MNIST digits at **h** at 1 epoch and **i** 104 epochs

To evaluate the potential of Janus MoSSe-based memory devices as neuromorphic systems, we implement the excitatory postsynaptic potential (EPSP) behavior of biological signaling (Fig. 4a). To mimic biological behavior in a memory device, we observed fluctuations in excitatory postsynaptic current (EPSC) induced by applied pulses. To realize short-term synaptic plasticity (STP), we examined the paired-pulse facilitation (PPF) property, an enhanced response following two consecutive stimuli [39]. We found that the PPF index (A_2_/A_1_), defined as the ratio of the EPSC (A_2_) to the second pulse to the EPSC (A_1_) to the first pulse, decreased as the pulse interval increased (Fig. 4b). This decreasing trend was fitted with a quadratic exponential function [40–42]:

5 $$PPF={C}_{0}+{C}_{1}\mathit{exp}\left(-\Delta t/{\tau }_{1}\right)+{C}_{2}\mathit{exp}\left(-\Delta t/{\tau }_{2}\right)$$

where *C*_0_ is the constant, and *C* and τ represent the facilitation magnitude and the relaxation time constants, respectively (${\tau }_{1}$: slow phase, ${\tau }_{2}$: rapid phase). The fitting results yielded τ values on the order of several hundred milliseconds, consistent with previously reported PPF behavior [43–45]. In addition, for long-term plasticity (LTP) with continuous stimulation, we applied 10 pulses (*t*_pulse_ = *t*_interval_ = 60 ms) (Fig. 4c). The Janus MoSSe device showed a rapid increase in EPSCs upon pulse application, with current retention behavior of at least 87% for 240 s duration. These characteristics were also tunable via pulse width and number, and these results demonstrate that the EPSC response can be tuned compositely to enable multistate realization (Fig. S11), which is a key requirement for in-memory computing using 2D-material-based memtransistor arrays [46].

In neuromorphic computing simulations, LTP/D is considered to be an important factor for neural network systems as it implements synaptic weight updates [47]. To investigate the LTP/D characteristics of the Janus MoSSe-based device, we applied 32 potentiation pulses (− 60 V) and 32 depression pulses (+ 30 V) successively (Fig. 4d). To assess stability under repeated operation, we monitored the EPSC responses during 100 LTP/D cycles consisting of a total of 6400 pulses. Throughout the cycles, the device exhibited stable operation with consistently reproducible weighted potentiation and inhibition (Fig. S12). Finally, we extracted values of device characteristics such as *NL* and dynamic range (*G*_max_/*G*_min_) from the LTP/D curves to perform simulations in an artificial neural network (see details in the Experimental Section). These properties remained stable over 100 cycles without significant degradation (Fig. S13). In addition to stability over cycles, LTP/D curves extracted from five different devices confirmed that there was no significant variation between devices (Fig. S14). Based on the obtained characteristics of Janus MoSSe devices, we performed training and inference on a multilayer perceptron (MLP)-based ANN for MNIST digit recognition (Fig. 4e). Figure 4f shows the parallelizable array circuit design used to implement the ANN weight connections in the simulation. Values such as *NL*, dynamic range, and variation were applied to the computation to reflect the characteristics of the device. Simulation results show that the recognition accuracy is close to the ideal device at 94.27% (Fig. 4g). Recognition simulations based on the characteristics of Janus MoSSe memory showed that it can reliably perform computations with 92.75% accuracy, even considering variations between devices and cycles. The confusion matrix for MNIST digit recognition showed random recognition in the first epoch, but as the learning progressed, it showed a clear diagonal pattern and achieved successful learning (Fig. 4h). Janus MoSSe memory was also able to perform stable simulations on convolutional neural networks (CNNs), which are based on more complex and higher-order computations (Fig. S15). Taken together, these results confirm that the Janus MoSSe-based parallel processing system can reliably support not only simple pattern recognition but also advanced computational tasks, highlighting its strong potential for neuromorphic computing.

## Conclusions

In conclusion, this study successfully demonstrates a 2D nonvolatile flash memory device utilizing Janus MoSSe as the floating gate. The intrinsic dipole moment originating from the structural asymmetry of Janus MoSSe was identified, through both theoretical calculations and experimental validation, to play a pivotal role in charge trapping and storage. This built-in asymmetry induces electron localization and strong charge–dipole interactions, resulting in superior device performance with a wide memory window, excellent retention, endurance exceeding 10^4^ program/erase cycles, and rapid charge-trapping dynamics. Optimization of the h-BN tunneling thickness enabled a favorable balance between charge injection efficiency and retention, even at reduced thicknesses, evidencing that the intrinsic polarity of Janus MoSSe fundamentally enhances memory operation. Furthermore, Janus MoSSe-based devices exhibited promising neuromorphic functionalities applicable to neural network learning and recognition. These findings provide valuable guidelines for designing ultrafast and reliable nonvolatile memories based on Janus TMDs. They further highlight the potential of these materials in next-generation flexible electronics and artificial intelligence hardware.

## Supplementary Information

Below is the link to the electronic supplementary material.

Supplementary file1 (DOCX 4370 KB)
