# Supplementary material for: Dipole-Driven Charge Trapping in Monolayer Janus MoSSe for Ultrathin Nonvolatile Memory Devices
Source: Nanomicro Lett. 2026 Jan 26;18:216. doi: 10.1007/s40820-026-02078-y (PMC12832604; doi:10.1007/s40820-026-02078-y)
Supplement: Supplementary file 1 — Supplementary file1 (DOCX 4370 KB) [file 40820_2026_2078_MOESM1_ESM.docx]

Supporting Information for

**Dipole-Driven Charge Trapping in Monolayer Janus MoSSe for Ultrathin Nonvolatile Memory Devices**

Eun Bee Ko^1†^, Junho Sung^2†^, Seon Yeon Choi^1^, Yasir Hassan^3^, Jeong-Ju Bae^3^, Jongseok Kim^3^, Hyun You Kim^3^, Eunho Lee^2^, Min Sup Choi^3^*, Hyun Ho Kim^1,4^*

^1^ School of Materials Science and Engineering, Kumoh National Institute of Technology, Gumi 39177, Republic of Korea

^2^ Department of Chemical and Biomolecular Engineering, Seoul National University of Science and Technology, Seoul 01811, Republic of Korea

^3^ Department of Materials Science and Engineering, Chungnam National University, Daejeon 34134, Republic of Korea

^4^ School of Materials Science and Engineering, Gwangju Institute of Science and Technology, Gwangju 61005, Republic of Korea

*Corresponding authors. E-mail: [kimhh@gist.ac.kr](mailto:kimhh@gist.ac.kr) (Hyun Ho Kim); [goodcms@cnu.ac.kr](mailto:goodcms@cnu.ac.kr) (Min Sup Choi)

**Supplementary Figures**

**
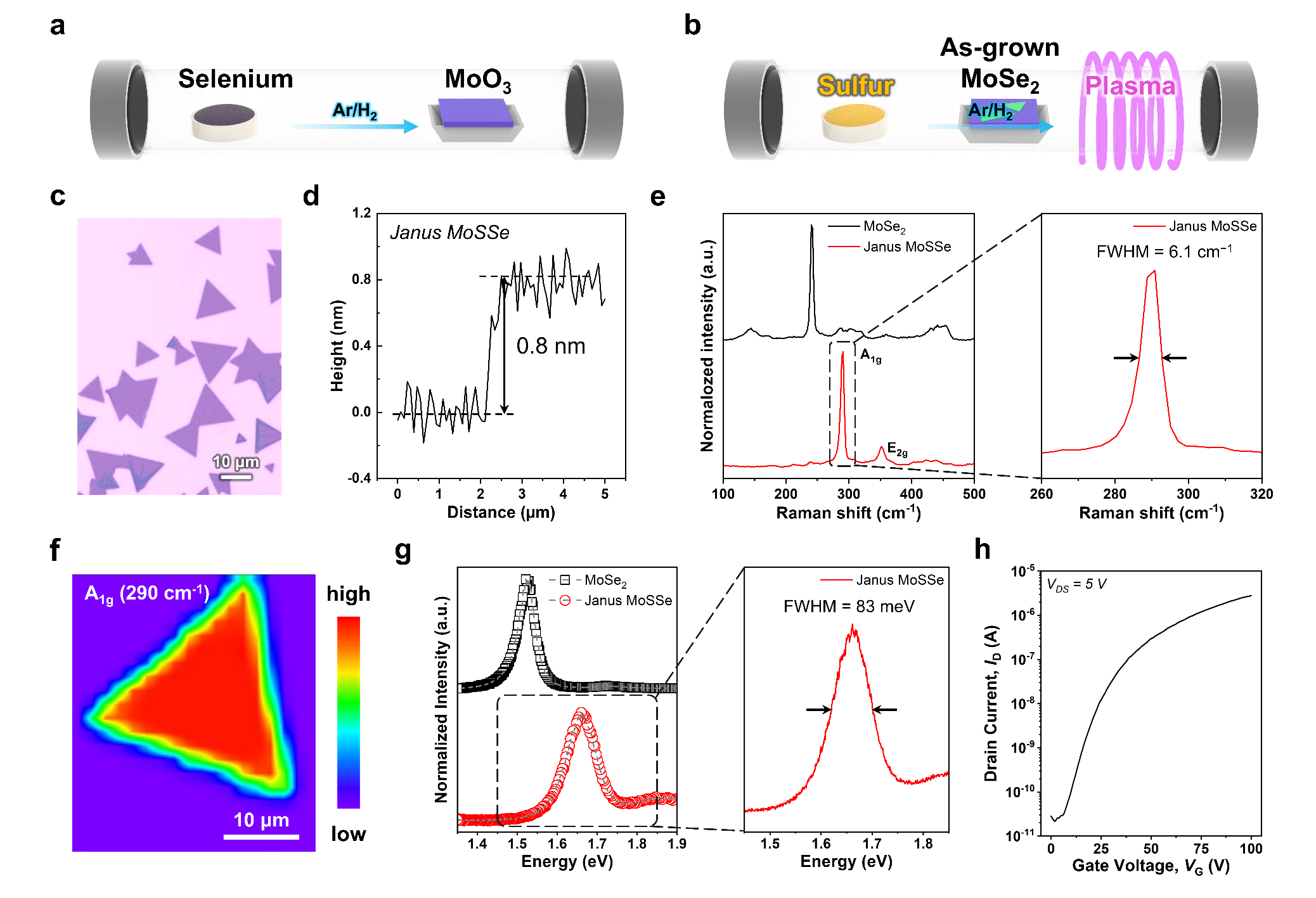
**

**Fig. S1 a** Schematic illustration of the APCVD process used for the growth of monolayer MoSe_2_. **b** Plasma-assisted sulfurization using PECVD to convert the as-grown MoSe_2_ into Janus MoSSe. **c** Optical microscopy (OM) image of the synthesized Janus MoSSe flakes. **d** AFM profile showing a thickness of ~0.8 nm. **e** Raman spectra of MoSe_2_ and Janus MoSSe. **f** Raman mapping of A_1g_ peak in Janus MoSSe. **g** PL spectra of MoSe_2_ and Janus MoSSe. **h** Transfer curve of a Janus MoSSe transistor, measured at a drain voltage of *V*_DS_ = 5 V.


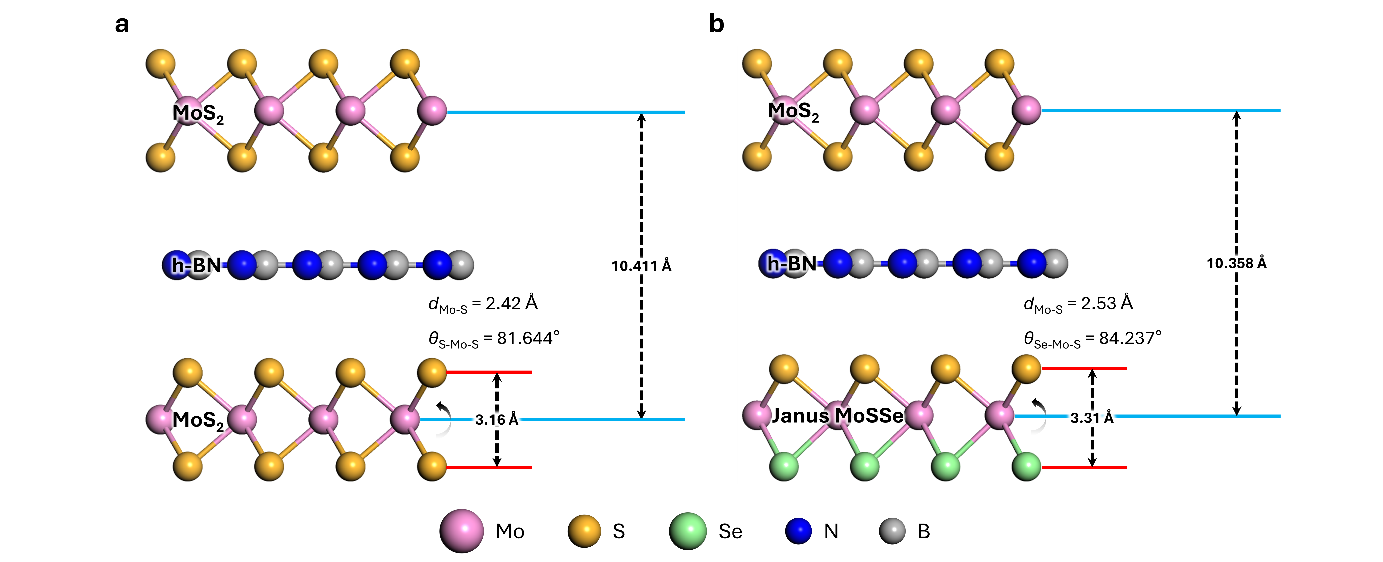


**Fig. S2** Atomistic models and key structural parameters of **a** MoS_2_/h-BN/MoS_2_ and **b** MoS_2_/h-BN/Janus MoSSe heterostructures. Interlayer distances, bond lengths (𝑑_Mo−S_​), and bond angles (𝜃_S−Mo−S_, 𝜃_Se−Mo−S_​) are indicated for each configuration, highlighting the structural modifications induced by the incorporation of the Janus layer.


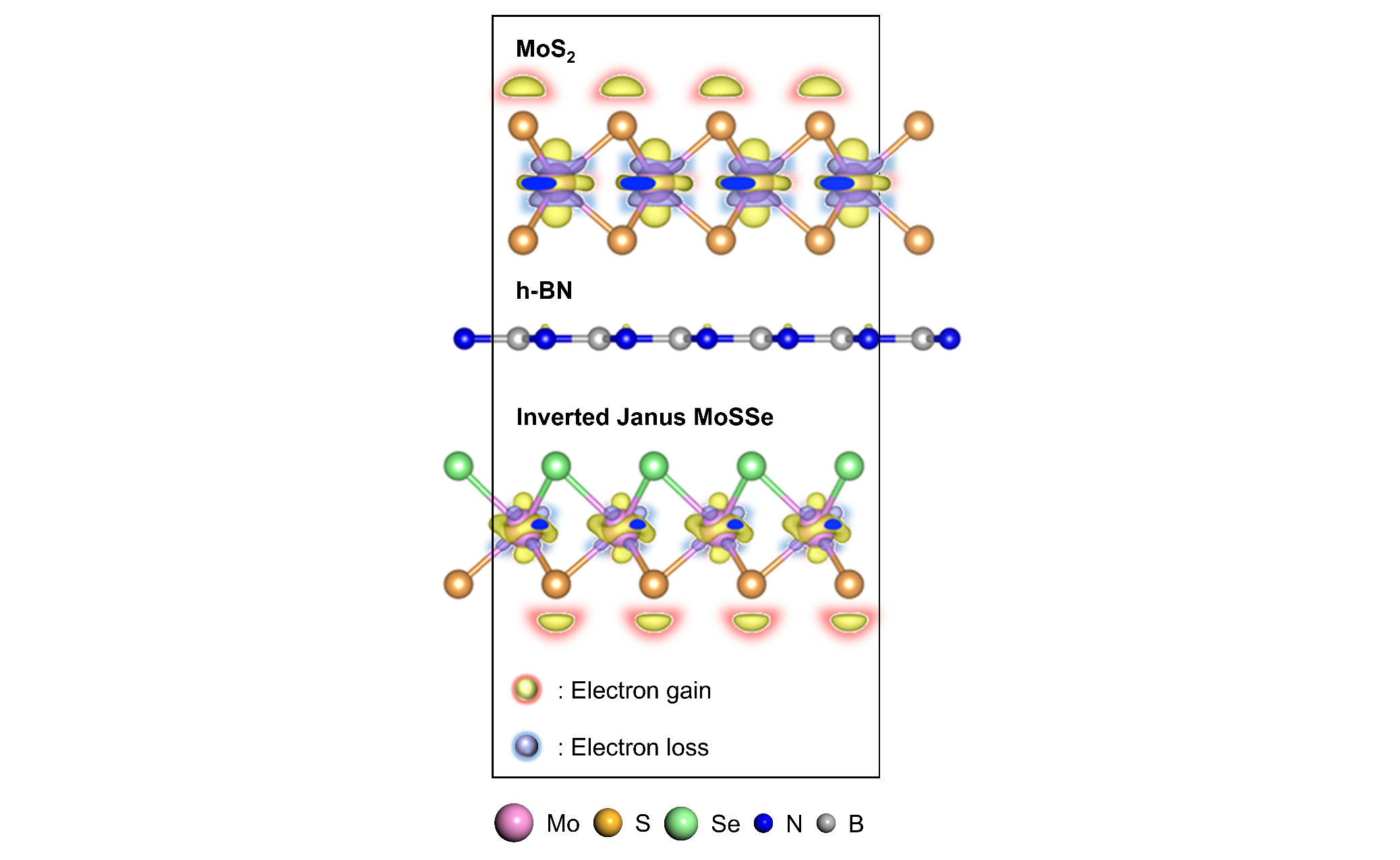


**Fig. S3** Inverted MoS_2_/h-BN/Janus MoSSe. Yellow and blue regions represent the area where electrons are localized and depleted, respectively


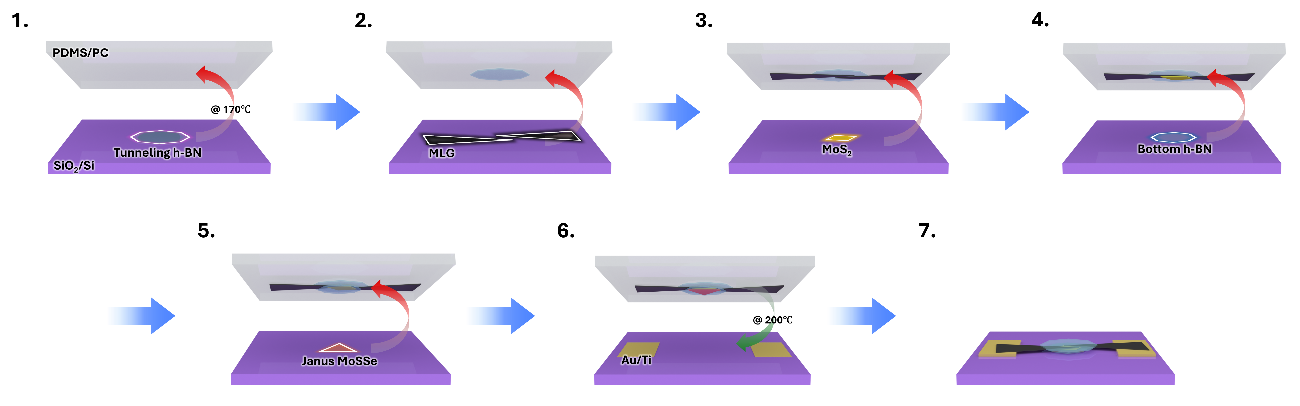


**Fig. S4** Schematic illustration of the fabrication process for the Janus MoSSe memory device. The constituent materials, including tunneling h-BN, multilayer graphene (MLG), MoS_2_, bottom h-BN, and Janus MoSSe, are sequentially picked up and transferred onto a substrate with pre-patterned Au/Ti electrodes


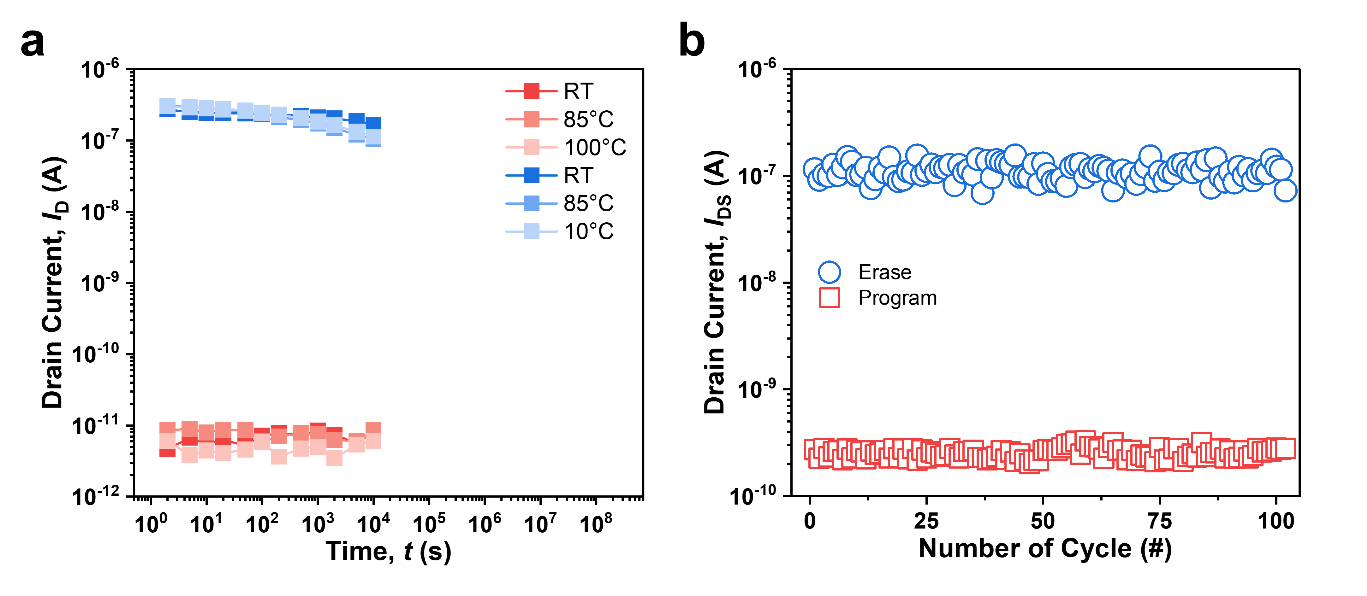


**Fig. S5** **a** Retention characteristics of the Janus MoSSe floating-gate memory device measured at room temperature, 85 °C, and 100 °C. **b** Endurance characteristics of the Janus MoSSe floating-gate memory device measured with 500 ns program/erase pulses for over 100 cycles


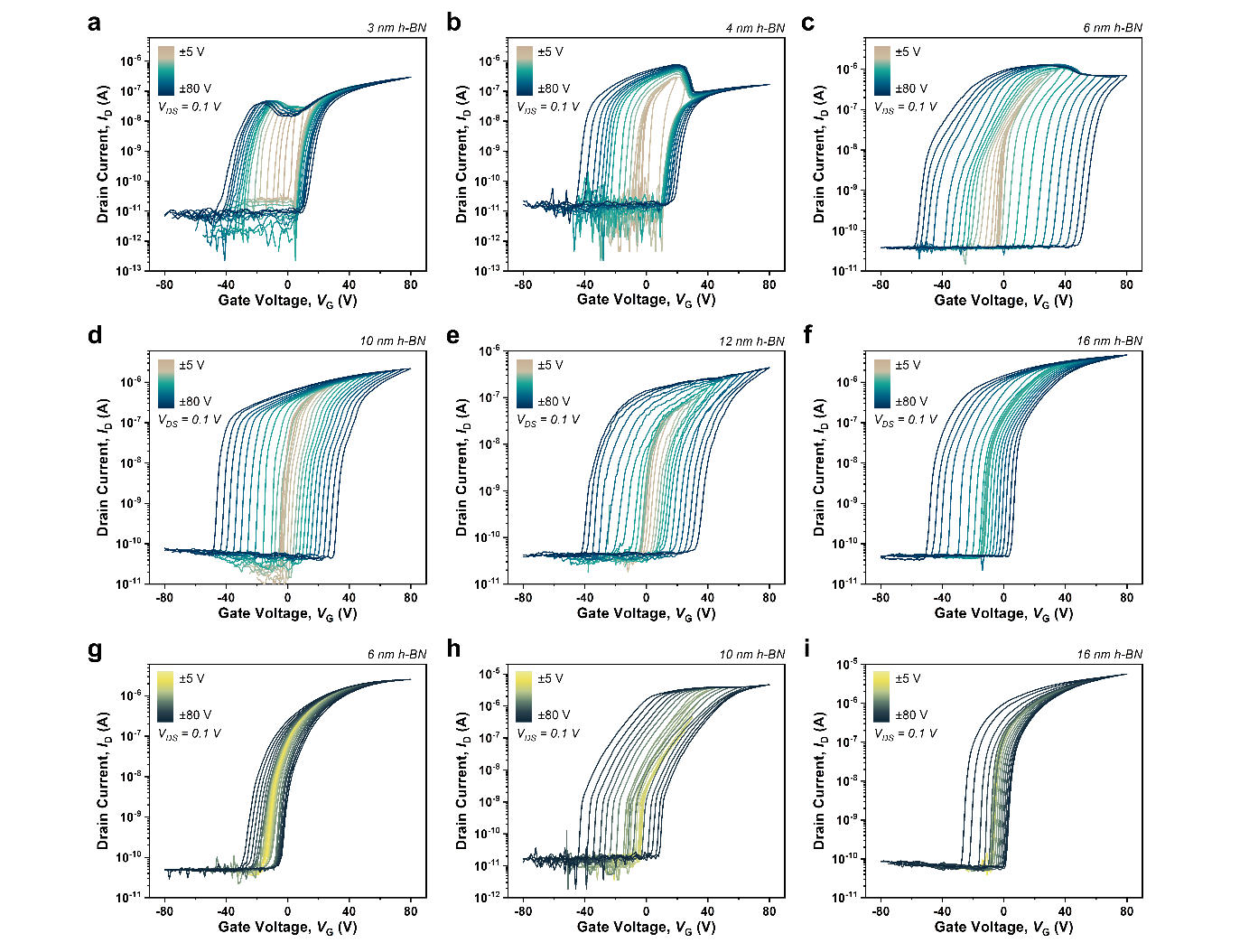


**Fig. S6** Transfer characteristics (𝐼_D_–𝑉_G_​) of memory devices with varying h-BN tunneling layer thicknesses. **a–f** Janus MoSSe floating-gate devices with h-BN thicknesses of 3, 4, 6, 10, 12, and 16 nm. **g–i** MoSe₂ floating-gate devices with h-BN thicknesses of 6, 10, and 16 nm


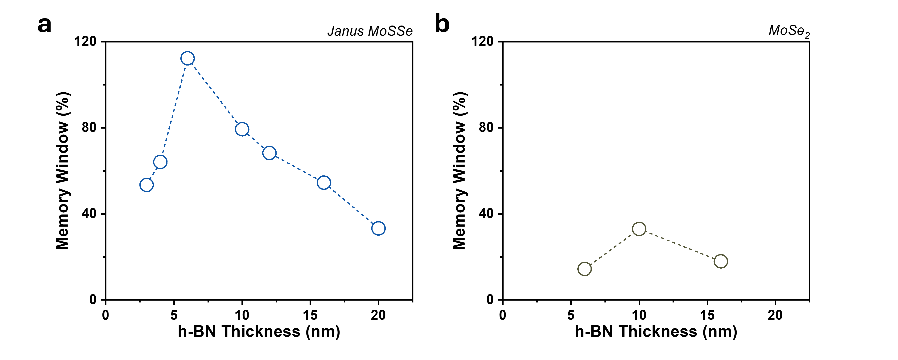


**Fig. S7** Summary of memory window variation as a function of *h*-BN thickness (2~20 nm) for both **a** Janus MoSSe and **b** MoSe_2_ devices

**Table S1** Benchmark table for comparing characteristics such as memory window and endurance based on floating gate, channel, and h-BN thickness

| Floating gate | Channel | Tunneling barrier | h-BN thickness (nm) | Memory window (%) | Long-term stability | Endurance | References |
| --- | --- | --- | --- | --- | --- | --- | --- |
| **Janus MoSSe** | **MoS_2_** | h-BN | **6** | **70** | **10^4^ s** | **10000 cycles** | **This work** |
| MLG | MoS_2_ |  | 4.6 | 13 | - | - | [6] |
|  | ReS_2_ |  | 6 | 63 | 10^4^ s | 1000 cycles | [16] |
|  | MoS_2_ |  | 6.1 | 28 | - | - | [6] |
|  | MoS_2_ |  | 7.5 | 55 | - | 1000 cycles | [6] |
|  | MoS_2_ |  | 8.4 | 36 | 1500 s | 1000 cycles | [10] |
|  | MoS_2_ |  | 10 | 36 | 8000 s | 4200 cycles | [17] |
|  | SnSe_2_ |  | 10 | 65 | 2000 s | 500 cycles | [S1] |
|  | InSe |  | 10 | 65 | 16.5 d | 2000 cycles | [7] |
|  | MoS_2_ |  | 10 | 60 | 1400 s | 110 cycles | [13] |
|  | MoS_2_ |  | 11.7 | 84 | 3800 s | 1000 cycles | [6] |
|  | MoTe_2_ |  | 13.3 | 82 | 300 s | 40 cycles | [S2] |
|  | Bi_2_O_2_Se |  | 13.7 | 79 | 1000 s | 500 cycles | [S3] |
|  | MoS_2_ |  | 13.8 | 64 | 10^4^ s | - | [S4] |
|  | ReS_2_ |  | 14.8 | 80 | 1000 s | 1000 cycles | [S5] |
| GaSe | InSe |  | ~7 | 63 | 3 d | - | [S6] |
| MoTe_2_ | WSe_2_ |  | 10.8 | 83 | 2000 s | 2000 cycles | [S7] |
|  | MoS_2_ |  | 8.8 | 86 | 3000 s | 1200 cycles | [S8] |
| Au | SnS_2_ |  | 23 | 66 | > 10^3^ | 15000 cycles | [S9] |

**
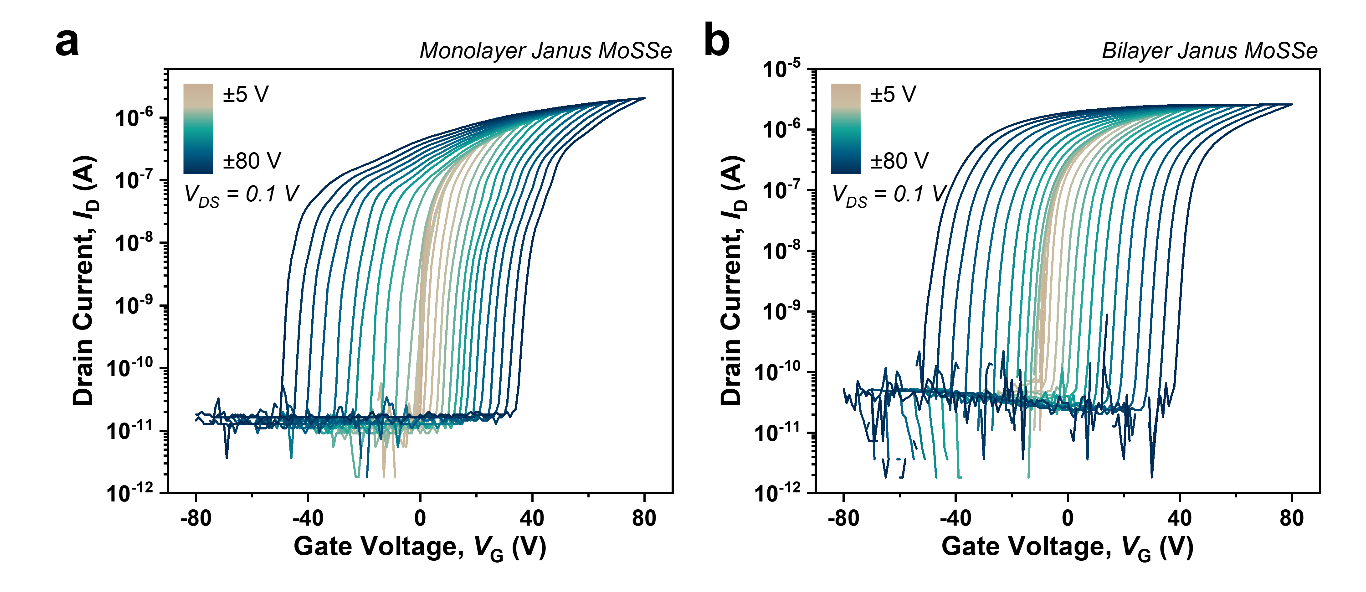
**

**Fig. S8** Transfer characteristics of floating-gate memory devices using **a** monolayer Janus MoSSe and **b** bilayer Janus MoSSe as the charge-trapping layer


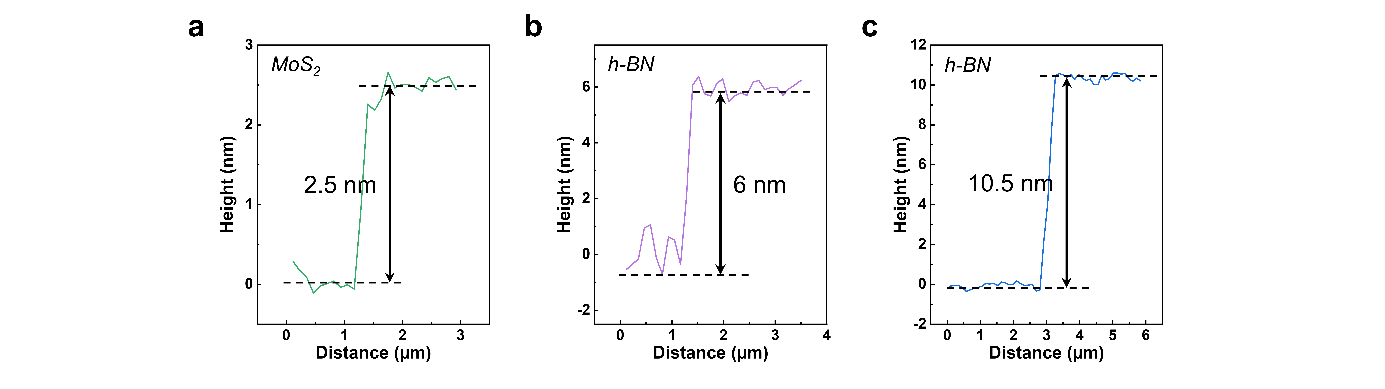


**Fig. S9** AFM height images of **a** MoS_2_ channel and **b, c** h-BN tunneling layers with thicknesses of 6 nm and 10 nm, respectively


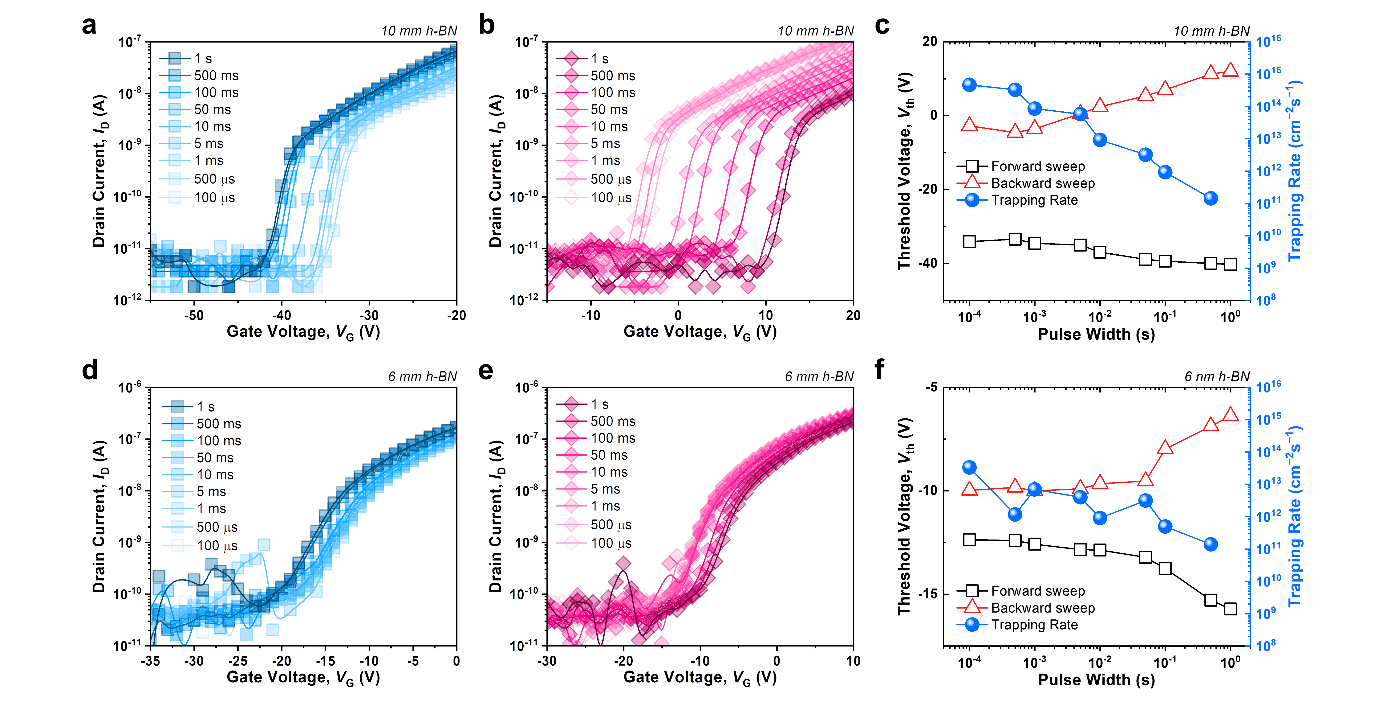


**Fig. S10** Pulse-width dependent transfer characteristics of MoSe_2_ floating-gate memory devices with different h-BN tunneling layer thicknesses: **a–c** device with 10 nm h-BN and **d–f** device with 6 nm h-BN. **a, d** Forward sweep; **b, e** backward sweep for various pulse widths; **c, f** extracted threshold voltage and charge trapping rate as a function of pulse width


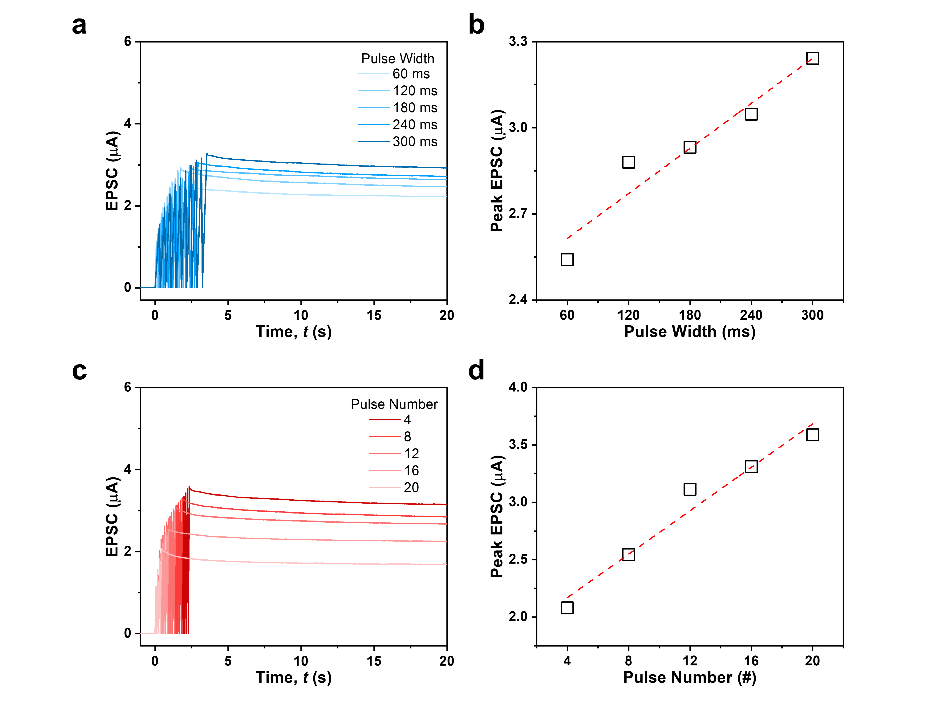


**Fig. S11** Changes in LTP characteristics as a function of **a** width and **c** number of pulses applied to Janus MoSSe devices. Variation of peak EPSC as a function of **b** pulse width and **d** number


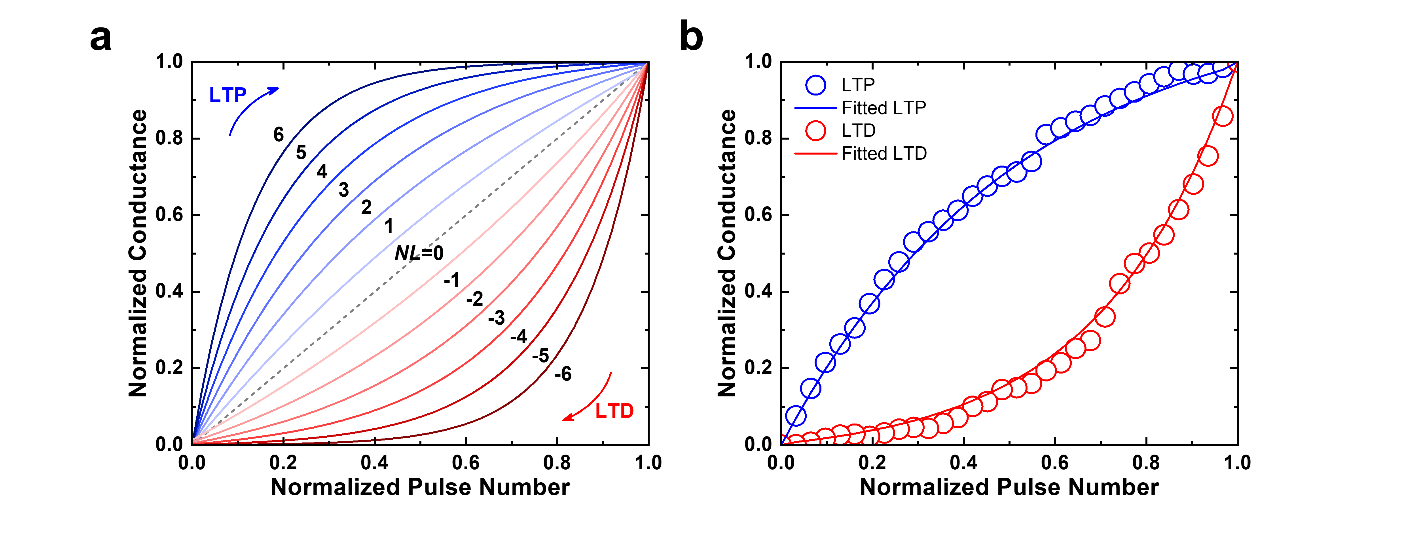


**Fig. S12** **a** Curves as a function of *NL* value used in the fitting of the LTP/D characteristic. **b** The change in normalized conductance measured in the Janus MoSSe device (symbol) and the curve fitted to the decreasing-increasing trend (line)


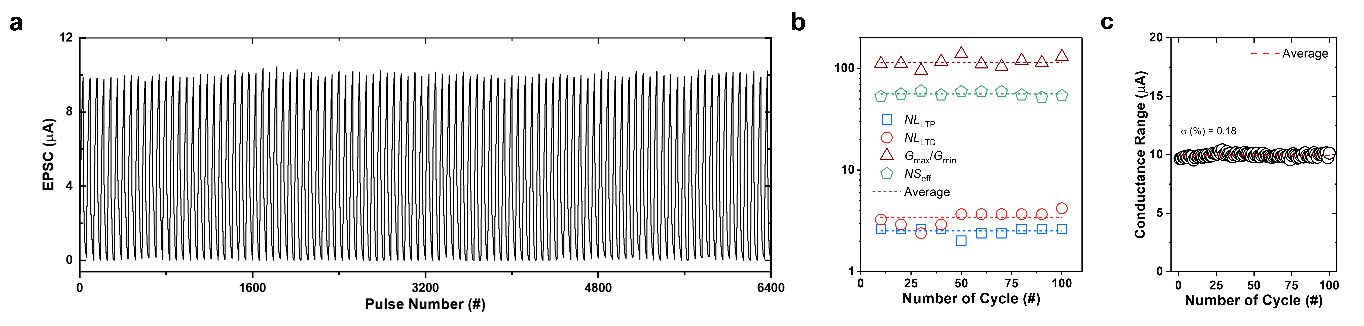


**Fig. S13** **a** LTP/D curve obtained by applying 6400 pulses to a Janus MoSSe device over 100 cycles. **b** Changes in *NL*_LTP/D_, *G*_max_/*G*_min_, and *NS*_eff_ values during 100 cycles of LTP/D measurements. **c** Variation of the conductance range defined as *G*_max_ − *G*_min_ for 100 cycles of LTP/D


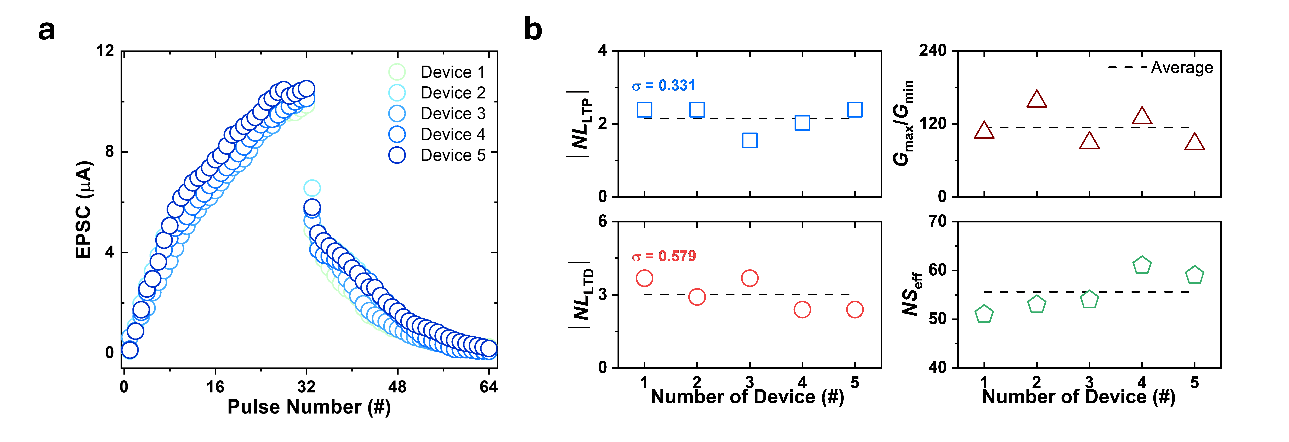


**Fig. S14** **a** LTP/D properties obtained from five different devices. **b** Variation of the characteristic parameters across devices


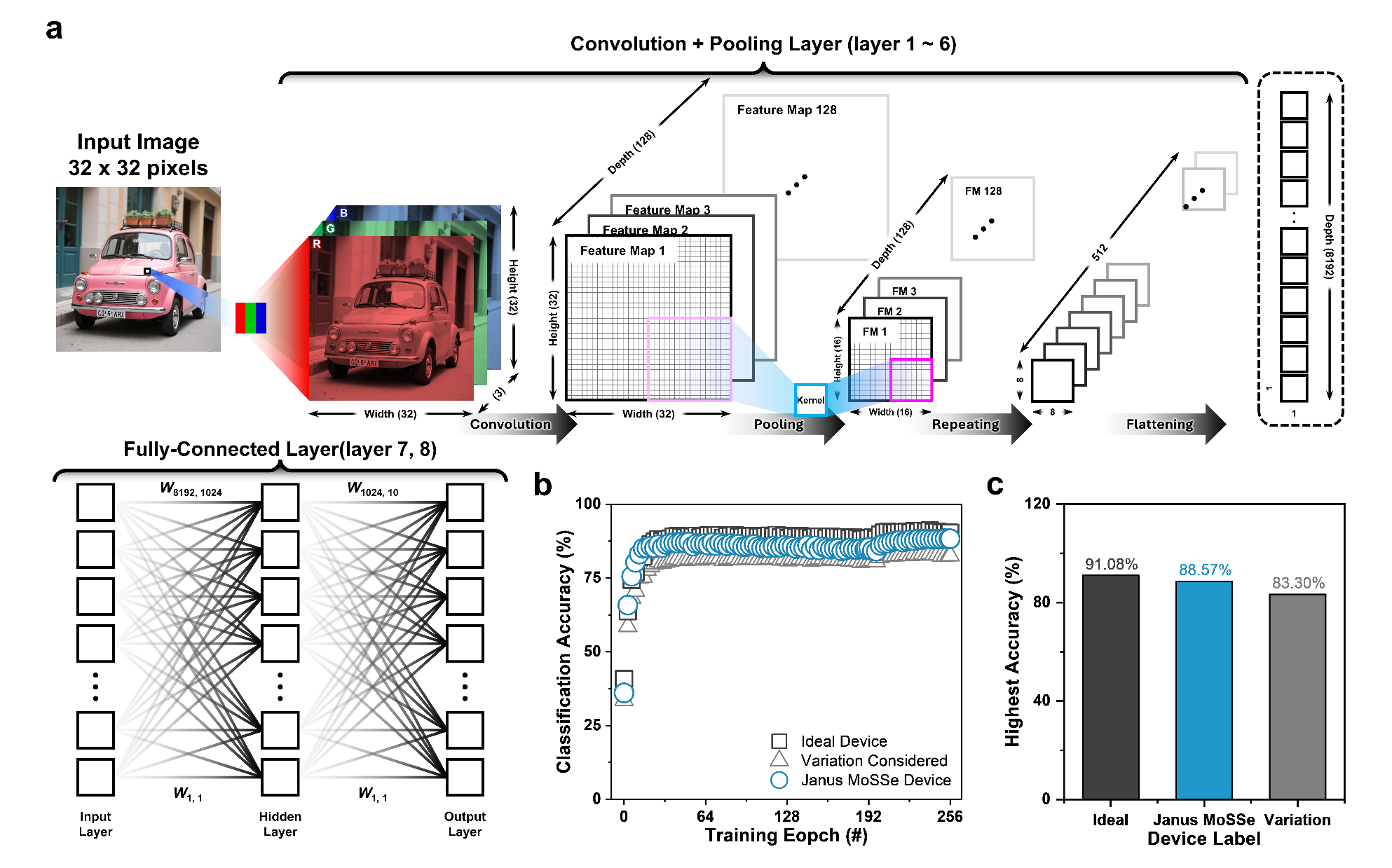


**Fig. S15 a** Schematic of a convolutional neural network with VGG-8 structure for CIFAR-10 data recognition simulation. **b** Recognition accuracy trend and **c** highest accuracy for ideal device, Janus MoSSe device and variation considered Janus MoSSe device over 256 epochs of training and inference

**Supplementary References**

1. G. Dastgeer, S. Nisar, A. Rasheed, K. Akbar, V. D. Chavan et al., Atomically engineered, high-speed non-volatile flash memory device exhibiting multibit data storage operations. Nano Energy. **119**(109106 (2024). https://doi.org/10.1016/j.nanoen.2023.109106
2. Y. Zhang, L. Wang, Z. Huang, W. Deng, X. Yan et al., Nonvolatile memory and neuromorphic devices based on the MoTe_2_/h-BN/graphene floating gate transistor. Appl. Mater. Today. **46**(102908 (2025). [https://doi.org/10.1016/j.apmt.2025.102908](https://doi.org/https://doi.org/10.1016/j.apmt.2025.102908)
3. L. Sun, Y. Xu, G. Huo, Y. Hou, W. Li et al., Multifunctional neuromorphic optoelectronic computing using all 2D floating-gate transistors. Nano Energy. **143**(111311 (2025). [/https://doi.org/10.1016/j.nanoen.2025.111311](https://doi.org/https://doi.org/10.1016/j.nanoen.2025.111311)
4. W. Huang, L. Yin, F. Wang, R. Cheng, Z. Wang et al., Multibit optoelectronic memory in top-floating-gated van der waals heterostructures. Adv. Funct. Mater. **29**(36), 1902890 (2019). <https://doi.org/10.1002/adfm.201902890>
5. W. Li, J. Li, T. Mu, J. Li, P. Sun et al., The nonvolatile memory and neuromorphic simulation of ReS_2_/h-BN/graphene floating gate devices under photoelectrical hybrid modulations. Small. **20**(30), 2311630 (2024). <https://doi.org/10.1002/smll.202311630>
6. X. Gong, Y. Zhou, J. Xia, L. Zhang, L. Zhang et al., Tunable non-volatile memories based on 2D InSe/h-BN/GaSe heterostructures towards potential multifunctionality. Nanoscale. **15**(35), 14448-14457 (2023). <https://doi.org/10.1039/D3NR02995F>
7. C. Yao, G. Wu, M. Huang, W. Wang, C. Zhang et al., Reconfigurable artificial synapse based on ambipolar floating gate memory. ACS Appl. Mater. Interfaces.**15**(19), 23573-23582 (2023). <https://doi.org/10.1021/acsami.3c00063>
8. Y. Xia, J. Zha, H. Huang, H. Wang, P. Yang et al., Uncovering the role of crystal phase in determining nonvolatile flash memory device performance fabricated from MoTe_2_-based 2D van der waals heterostructures. ACS Appl. Mater. Interfaces. **15**(29), 35196-35205 (2023). <https://doi.org/10.1021/acsami.3c06316>
9. M.A. Khan, S. Yim, S. Rehman, F. Ghafoor, H. Kim et al., Two-dimensional materials memory devices with floating metal gate for neuromorphic applications. Mater. Today Adv. **20**(100438 (2023). <https://doi.org/10.1016/j.mtadv.2023.100438>
